# Comparative Genomic Analysis of the 2016 *Vibrio cholerae* Outbreak in South Korea

**DOI:** 10.3389/fpubh.2019.00228

**Published:** 2019-08-16

**Authors:** Sung-min Ha, Mauricio Chalita, Seung-Jo Yang, Seok-Hwan Yoon, Kyeunghee Cho, Won Keun Seong, Sahyun Hong, Junyoung Kim, Hyo-Sun Kwak, Jongsik Chun

**Affiliations:** ^1^School of Biological Sciences, Seoul National University, Seoul, South Korea; ^2^ChunLab Inc., Seoul, South Korea; ^3^Interdisciplinary Program in Bioinformatics, Seoul National University, Seoul, South Korea; ^4^Center for Laboratory Control of Infectious Diseases, Korea Centers for Disease Control, Cheongju-si, South Korea; ^5^Centers for Disease Control and Prevention, Korea Centers for Disease Control, Cheongju-si, South Korea; ^6^Food Microbiology Division, National Institute of Food and Drug Safety Evaluation, Chungcheongbuk-do, South Korea

**Keywords:** cholera, *Vibrio cholerae*, genome sequencing, molecular epidemiology, comparative genomics analysis

## Abstract

In August 2016, South Korea experienced a cholera outbreak that caused acute watery diarrhea in three patients. This outbreak was the first time in 15 years that an outbreak was not linked to an overseas source. To identify the cause and to study the epidemiological implications of this outbreak, we sequenced the whole genome of *Vibrio cholerae* isolates; three from each patient and one from a seawater sample. Herein we present comparative genomic data which reveals that the genome sequences of these four isolates are very similar. Interestingly, these isolates form a monophyletic clade with *V. cholerae* strains that caused an outbreak in the Philippines in 2011. The *V. cholerae* strains responsible for the Korean and Philippines outbreaks have almost identical genomes in which two unique genomic islands are shared, and they both lack SXT elements. Furthermore, we confirm that seawater is the likely source of this outbreak, which suggests the necessity for future routine surveillance of South Korea's seashore.

## Introduction

Cholera is an infectious disease that causes severe and acute diarrhea. Due to its severity, patients often experience hypovolemic shock, acidosis, and even death (1). The disease is transmitted through the consumption of *Vibrio cholerae*, a bacterial species found in seafood and polluted water. Although much is known about cholera, it is one of many infectious diseases that has still not been eradicated and repeated outbreaks continue to occur around the globe, especially in developing countries. The outbreak in Haiti in 2010 was one of the worst cholera outbreaks in recent history and resulted in 665,000 cases and 8,183 deaths. Owing to its severity, multiple investigations have been conducted to elucidate the origin and the transmission of this outbreak (2–4).

*V. cholerae* strains are classified by according to the O antigen serotypes, and there are currently >200 serogroups (5). Among these serogroups, O1 was the only serogroup that had caused major cholera pandemics until the emergence of O139 (6). Interestingly, the O1 serogroup has two major biotypes; classical and El Tor. Although classical *V. cholerae* has caused major outbreaks in the sixth pandemic era, it has not caused an outbreak since 1961 (7). On the other hand, the El Tor biotype was discovered in a quarantine station in Sinai in 1905 and obtained its notoriety from a global outbreak started in Indonesia in 1961, where it quickly overtook the classical biotype which opened the door for the seventh pandemic (8). The classical biotype has since lost its dominance over El Tor, but in recent years, variant strains possessing genotypes of Classical biotype in the CTX prophage region with the El Tor genome backbone have been reported (9–12).

Since the latest outbreak in 2001 (13), no local cholera cases have been reported in South Korea, except for patients who had traveled overseas to cholera endemic areas. In August 2016, for the first time in 15 years, an outbreak of cholera occurred when three local citizens were infected with *V. cholerae*. These patients were immediately quarantined following diagnosis of *V. cholerae* infection. The Korea Centers for Disease Control and Prevention (KCDC) used pulsed-field gel electrophoresis (PFGE) to find the epidemiological links and the source. The PFGE have shown that the strains isolated from these three patients, and an isolate from a seawater sample near the outbreak share the same PFGE patterns (14). In this study, we further investigated the molecular epidemiology of these isolates using whole genome sequencing, and we performed comprehensive comparative genomic (CG) analysis to elucidate the possible source, phylogenetic relationships, and their CTX prophage phenotype using molecular evidence. Through extensive CG analysis, we were able to confirm that the 2016 Korean isolates share a common ancestor with the *V. cholerae* strains that were responsible for the 2011 outbreak in the Philippines (12).

## Materials and Methods

### Genome Sequencing, Assembly, and Annotation

The KCDC provided genomic DNA of the four isolates (three from patients and one from a seawater sample). The detailed history of these strains was given in the previous study (14). DNA libraries were prepared using the TruSeq DNA library kit (Illumina, San Diego, CA, USA). The whole genome sequencing was performed using the MiSeq 250 paired-end system, according to the manufacturer's instructions. The sequencing reads were assembled using SPAdes 3.9.1 (15). Samples were tested for contamination by comparing different copy of 16S rRNA in the genome using ContEst16S tool v1.0 (16) and genome-based species identification was performed by the TrueBac ID system (v1.92, DB:20190603) [https://www.truebacid.com/; (17)] according to the algorithm proposed by Chun et al. (18). Each genome was matched against *V. cholerae* type strain genome to ensure that ANI is above the threshold value (≥ 95%).

Gene-finding was performed using Prodigal v2.6.3 (19) and gene annotation was conducted by homology search (USEARCH v8.1.1861) against the EggNOG v4.1 (20), SEED 2015-12-10 (21), Swiss-Prot [version 2015-12-10; (22)] and KEGG databases [version 2018-10-01; (23)] with following parameters; -accel 1.0 -evalue 1.0E-5 -maxaccepts 1. In addition, antibiotic resistance genes were identified using the Resistance Gene Identifier (RGI) v4.2.0 tool provided by the CARD database v3.0.2 (24).

### Searching for Phylogenetic Neighbors in the *Vibrio cholerae* Genome Database

To find the closest phylogenetic neighbors in the Genbank genome database, we developed a Single Nucleotide Variant (SNV)-based search algorithm. First, we generated a core gene set from 32 representative complete genomes (Table S1) belonging to *V. cholerae* using the Roary v3.12.0 pipeline (25). The resultant collection of core genes were concatenated to create a Species-specific Reference Genome (SRG), namely for *V. cholerae*. This artificially generated genome sequence was then used to detect the SNVs from 789 high-quality *V. cholerae* genome sequences in the EzBioCloud database (26), and the genome of the Korean *V. cholerae* isolates in this study (Table S2). Using SRG as a reference, a pairwise SNV analysis was performed using the nucmer and show-snps script in MUMmer v3.23 tool (27) against all 789 genomes and Korean isolates.

Genomes which are phylogenetically related to the Korean isolates were identified by analysis of pairwise genome similarity, which was calculated by comparing SNVs against the SRG. Alternatively, a genome-wide phylogenetic tree was generated from the SNV bases that were compiled from the calculated SNVs. The maximum likelihood method implemented in the FastTree tool v2.1 (28) was used for tree-making with the default parameters. The final dataset, containing the Korean isolates and their phylogenetic neighbors, was created using both similarity values and the topology of a phylogenomics tree based on SNVs.

### Genome-Wide Phylogenetic Analysis

The SNVs of genome sequences in the final dataset were calculated using the MUMmer v3.23 program (27) with isolate KorC1, the strain obtained from the first patient, as the reference genome sequence. A multiple sequence alignment was compiled from the detected SNVs and a maximum likelihood phylogenetic tree was inferred using RAxML v8.2.11 with 1,000 bootstrap re-samplings and GTRCAT as a model for nucleotide (29).

To perform whole genome multilocus sequence typing (wgMLST), the set of core genes that were detected in the process of creating the SRG was used. We generated the hidden Markov models (HMMs) for each of the resultant core genes using MAFFT v7.3.10 (30) and nhmmer v3.1b2 tools. The core genes were identified using an HMM-based search, and then used to assign unique allele numbers for each gene. The in-house JavaScript was coded for Kruskal's algorithm to infer a wgMLST-based minimum spanning tree (31).

### Comparative Genomics Based on Gene Contents

The gene content-based comparative genomic analysis was performed as described by Chun et al. (32). The USEARCH v8.1.1861 (33) tool was used for bidirectional protein sequence searches. Only genomic regions with five or more consecutive genes in a contig and appearance of the same patterns in different strains were considered genomic islands (GIs), when they are thought to be laterally transferred.

### Variants in CTX φ

The *ctxB1*(the classical allele), *ctxB2* (El Tor allele), *rstR1*(classical allele), *rstR2*(El Tor allele) were obtained from two genomes (GCA_000621645.1/ATCC 14035 O1 Classical and GCA_000006745.1/N16961 O1 El Tor; Table S3). BLAST v2.2.30+ was used to search for different alleles in Korean isolates. After the search, EzBioCloud's genome browser was used to locate the CTXφ prophage with the structure proposed by Davis et al. [(34); Figure S1].

## Results

### Genomic Features of Korean *V. cholerae* Isolates

The draft genome sequences of the four isolates in this study were assembled to give 61–99 contigs and have a total length of 3.96–3.99 Mbp, with the average G+C content of 47.5 mol% (Table 1). Subsequent genome annotation showed that they have an average of 3,483 coding sequences (CDSs). The *ctxB* and *rstR* genes, which belong to the *CTX*φ and RS1 region respectively, were selected as genetic markers to differentiate between the *CTX*φ genotypes (35, 36). A single CTXφ region, which include the core and RS2 regions, was found in all isolates. In the core region, *ctxB1* (the classical allele) that contains histidine and threonine in position 39 and 68, respectively, was found along with *ctxA, zot, ace, orfU*, and *cep* (37). Due to the incompleteness of the genome assemblies, only partial fragments of RS1, which is absent in the classical biotype strain, and RS2 regions were recovered, and sufficient to confirm that Korean isolate contains a hybrid form of CTXφ prophage (34, 38). The presence of the *rstC* gene suggests the presence of an *RS1* region, and both types of *rstR* (classical and El Tor) were found.

**Table 1:** Patient information and genomic properties of *V. cholerae* isolates.

	**Patient #1**	**Patient #2**	**Patient #3**	**Sea water**
Gender	Male	Female	Male	–
Source	Fecal	Fecal	Fecal	Jangmuk
Age	59	73	63	–
Onset of diarrhea	2016-8-9	2016-8-15	2016-8-21	–
First admission day	2016-8-11	2016-8-17	2016-8-23	–
Duration of diarrhea	8 days	9 days	9 days	–
Underlying symptoms	Angina Pectoris	Hypertension stroke	Hypertension	–
Date of seafood consumption	2016-8-7,2016-8-8	2016-8-14	2016-8-18	–
Type of seafood consumed	Seabass, sea squirt, abalone, crab	Japanese Spanish mackerel	Assorted raw seafood^*^	–
Genome size(bp)	3,958,379	3,957,889	3,974,533	3,960,629
No. of contigs	84	84	77	61
N50(bp)	377,211	377,211	636,791	636,791
GC ratio (%)	47.5	47.5	47.5	47.5
No. of CDSs	3,476	3,476	3,495	3,489
16S rRNA similarity to CECT 514^T^ (%) (Accession X76337)	99.7	99.7	99.7	99.7
Average nucleotide identity to ATCC 14035 (%) (Accession GCA_000621645.1)	99.3	99.3	99.3	99.3

* *Assorted raw seafood include horse mackerel, flatfish, rockfish, sea squirt, abalone, shrimp, sea cucumber, octopus, and squid*.

### Genome-Wide Phylogenetic Analysis

Using an SNV-based search, a total of 17 genome sequences in the EzBioCloud database were selected for the final dataset along with the four Korean isolates. These genomes showed the highest sequence similarities and were placed closely in the SNV-based phylogenetic tree. Notably, three strains that had been associated with the 2011 Philippines outbreak were also included.

**Figure 1:**
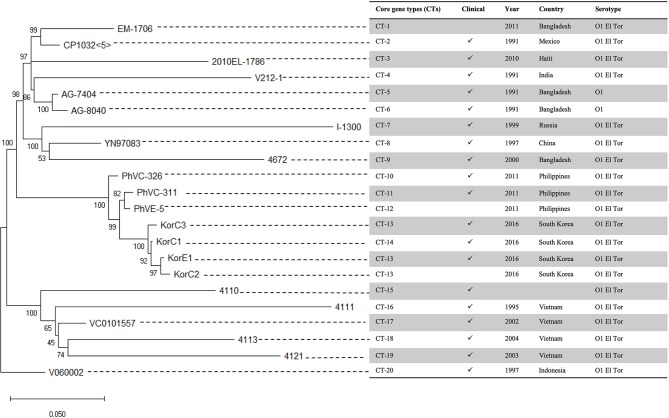
Maximum likelihood phylogenetic tree of Korean isolates and related strains with single nucleotide variant (SNV) data. The tree was generated using RAxML. The scale bar indicates substitution rate per site. Analysis of wgMLST data shows that 3 Korean strains (KorC2, KorC3, and KorE1) shared the same core gene type, and that the KorC1 strain only differs by one hypothetical protein.

A total of 976 variable positions were identified from the SNVs detected in the final dataset. Intriguingly, the maximum likelihood phylogenetic tree revealed that both of the strains from Korea and the Philippines form a monophyletic clade. This was supported by a 100% bootstrap value (Figure 1). The tree topology within this Korea/Philippines clade indicates that all *V. cholerae* outbreaks in Korean and in the Philippines share a common ancestor.

**Figure 2:**
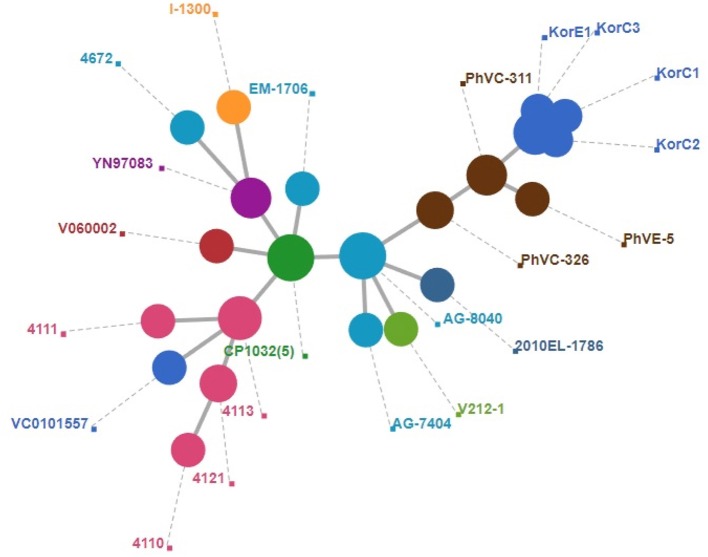
Minimum spanning tree of wgMLST based on 2,340 core genes of *V. cholerae*. A cluster of blue circle depicts Korean outbreak strains and nearest yellow circles indicate the strains that caused the 2011 Philippines outbreak.

A total of 2,341 core genes were identified by the Roary v3.12.0 tool and used as house-keeping genes in wgMLST analysis. Among the genomes included in the final dataset, 424 genes showed different sequence types. The wgMLST-based minimum spanning tree is given in Figure 2 which also indicates that Korean isolates were descendants of the Philippines outbreak strains in 2011. All the Korean strains showed exactly same core gene types except that the KorC1 strain which showed a different gene type in one gene VC2032, which encodes for a hypothetical protein. In contrast, all of the strains from the Philippines were more distinct from one another.

### Comparative Genomics

**Figure 3:**
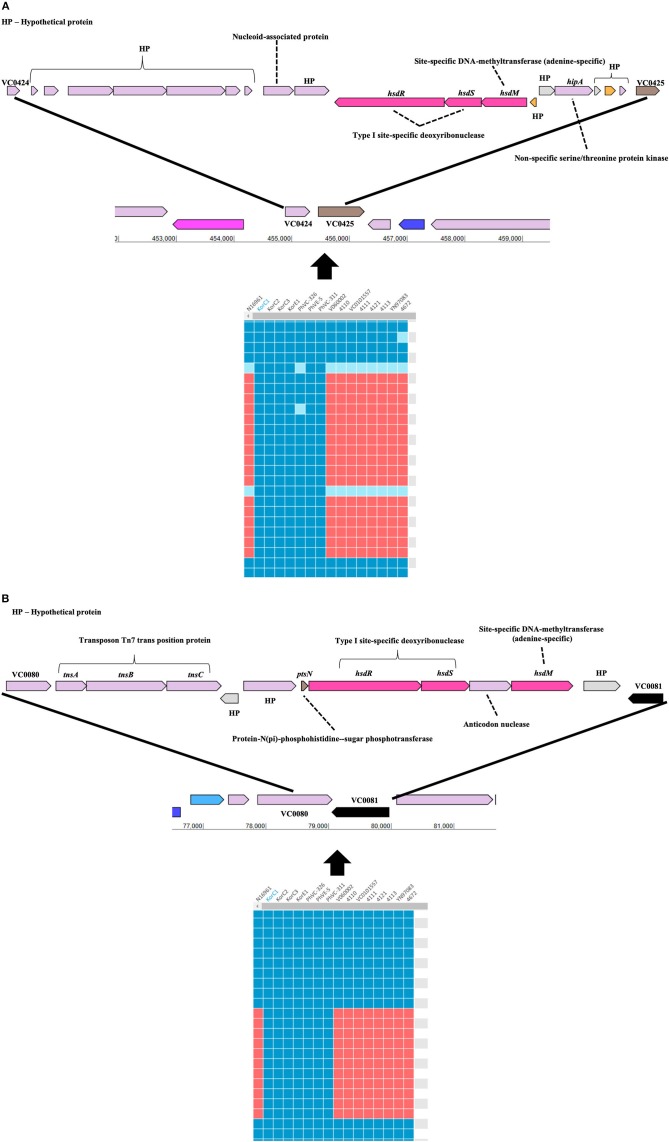
Genomic islands that are uniquely present in both Korean and Phillipinnes outbreak strains. Boxes in blue and red indicate the presence and absence, respectively, of the orthologous gene in the genome to genome comparison. HP, hypothetical protein. **(A)** Genomic island 1 is located between VC0424 and VC0425. **(B)** Genomic island 2 between VC0080 and VC0081. Both genomics islands contain Type I specific deoxyribonucleases and adenine-specific DNA-methyltransferase.

Gene-content based comparative genomic analysis revealed functional similarities and differences, as well as lateral gene transfer events among genomes in the final dataset (Figure 1). Two GIs were identified between the Korea/Philippines clade and the remaining *V. cholerae* strains from various countries. Figure 3 depicts the genetic organization of two GIs, named as genomic islands of Korea/Philippines 1 (GI-KP1) and genomic islands of Korea/Philippines 2 (GI-KP2), that were only present in the Korea/Philippines clade. The GI-KP1 island harbors the genes related to the type I modification system and truncated *Vibrio* pathogenicity island 2 (VPI-2), which had been reported earlier (12). The GI-KP2 island consists of 13 genes encoding transferases, kinases, nucleases, and hypothetical proteins. In contrast, all strains in the Korea/Philippines clade lacked the SXT element that all other *V. cholerae* isolates possess.

Pairwise SNV analysis revealed that 15–25 and 18–26 substitutions among the 2016 Korean and 2011 Philippines isolates, respectively. Higher pairwise substitutions were found between Korean and Philippines isolates, ranging from 26–45 with an average of 36.3 substitutions. The detailed SNV analysis is given in Table 2.

**Table 2:** Pairwise nucleotide differences among strains belonging to the 2011 Philippines and 2016 Korea outbreaks.

**Used as reference genome**	**KorC1**	**KorC2**	**KorC3**	**KorE1**	**PhVC-326**	**PhVE-5**	**PhVC-311**	**YN97083**	**V060002**
KorC1	-	15	19	23	45	41	37	110	113
KorC2	15	-	25	21	40	43	42	115	112
KorC3	21	27	-	20	36	31	29	116	95
KorE1	22	20	17	-	26	31	27	113	98
PhVC-326	45	40	36	26	-	26	18	83	79
PhVE-5	45	43	35	35	26	–	14	86	89
PhVC-311	37	42	30	28	18	12	–	93	89
YN97083	110	114	116	113	83	88	92	–	65
V060002	116	116	106	106	81	86	90	64	–

*MUMmer tool was used to calculate single nucleotide variants (SNVs)*.

Through the extensive analysis of SNV data, five non-synonymous mutations were found that are specific to Korean outbreak strains. Among these mutations, two of them were on uncharacterized protein and remaining three genes were annotated as *ftsI, prfC*, and *edd* (Table 3).

**Table 3:** SNVs from Korea outbreak strains that are distinct from the Philippine strains.

**SNV contig #: position**	**Gene function**	**Gene name**	**SNV**	**Substitution**
34: 245316	Cell division protein [Peptidoglycan synthetase]	*Ftsl*	G -> A	A553V
12: 51506	Peptide chain release factor3	*prfC*	A -> G	D35G
4: 129876	Phosphogluconate dehydratase	*edd*	. -> G	Insertion
67: 104132	Uncharacterized protein	–	T -> C	D75G
69: 111605	Uncharacterized protein	–	C -> T	G142D

*Data shown here only include shared SNV bases in all Korean strains that cause non-synonymous mutations from the Philippines strains*.

*SNV position is with reference to the PhVC-311 genome.

### Interactive Web Site for Comparative Genomics

The gene content-based comparative genomics and other results can be accessed at https://www.ezbiocloud.net/cg/vcholerae-kor2016 with an interactive user interface.

## Discussion

In this study, we investigated the epidemiology and genome-based characterization of the 2016 cholera outbreak in Korea using whole genome sequencing. Unlike the previous cholera outbreaks in South Korea over the last 15 years, the patients in this outbreak had no record of overseas travel. All three patients had consumed local seafood in the same county (within 20 km), and the toxin-positive *V. cholerae* strain was isolated from nearby seawater (14). In addition to their geographical association, both SNV-based phylogeny (Figure 1) and wgMLST (Figure 2) suggest that all Korean isolates from patients and seawater form a monophyletic clade with only 15–27 substitutions, thereby suggesting a strong epidemiological link. The fact that all Korean isolates from 2016 come from the same source was confirmed by wgMLST in which all genomes showed 0–1 allele differences out of 2,341 core genes.

In an earlier study by Kim et al. (14), PFGE was used for molecular epidemiology of this outbreak in which three patient isolates showed identical PFGE patterns and the seawater isolate differs only slightly with 97% identity. The authors concluded that the possible source of the outbreak was seawater given the result of PFGE typing, as well as the unusually favorable environmental conditions for *V. cholerae* that existed in 2016. Our genome-based study agrees with previous findings and also provides further epidemiological insights into the 2016 Korea outbreak, by comparing our large genome database, holding 789 strains of *V. cholerae* isolated from various countries and in different years.

In this study, we were able to find the epidemiological link between the Korea outbreak to strains that caused a *V. cholerae* outbreak in the Philippines in 2011 (12). This relationship is supported by both SNV-based phylogeny and wgMLST. Gene-content based comparative genomic analysis provides more supporting evidence for close relatedness of isolates from the two countries.

In conclusion, we have demonstrated the utility of whole-genome sequencing for the epidemiological study of *V. cholerae* outbreaks. Using a combination of phylogenomic and comparative genomic methods, we show that the three Korean cases are related to the *V. cholera* lineage associated with the 2011 Philippines outbreak. The case presented here demonstrates the need for ever more comprehensive genome sequence databases to pave the way for improved global monitoring of important pathogens such as *V. cholerae*.

## Data Availability

The datasets generated and analyzed for this study can be found in the EBI website at http://www.ebi.ac.uk/ena/data/view/PRJEB29273 and EzBioCloud at https://www.ezbiocloud.net/cg/vcholerae-kor2016, respectively.

## Author Contributions

The idea for this study was conceived by WS, SHo, H-SK, and JC. Sample collection and DNA extraction was performed by WS, SHo, JK, and H-SK. Genome sequencing and all other experiments were conducted by KC and S-JY, Reference searching, analyzing genomic data, and writing a draft of the manuscript scheme were done by SHa. Phylogenomic trees were reconstructed by S-HY, SHa, and MC, with applying the mechanisms and proposed idea. The manuscript was written mainly by SHa and JC.

### Conflict of Interest Statement

SHa, MC, S-JY, S-HY, KC, and JC are the employees of ChunLab, Inc., the developer of the EzBioCloud and TrueBac ID services. The remaining authors declare that the research was conducted in the absence of any commercial or financial relationships that could be construed as a potential conflict of interest.
