# Supplementary material for: Comparative Genomic Analysis of the 2016 Vibrio cholerae Outbreak in South Korea
Source: Front Public Health. 2019 Aug 16;7:228. doi: 10.3389/fpubh.2019.00228 (PMC6707086; doi:10.3389/fpubh.2019.00228)
Supplement: Supplementary file 1 [file Data_Sheet_1.docx]

Supplementary Material

# Supplementary table(s)

**Table S1.** List of genomes that was used for constructing the Species-specific Reference Genome (SRG)

| **NCBI accession** | **Taxon name** | **Serotype** | **Strain name** | **Genome size (bp)** | **Country** | **Year** |
| --- | --- | --- | --- | --- | --- | --- |
| GCA_000195065.1 | *Vibrio cholerae* | O1 | LMA3984-4 | 3,738,715 | Brazil |  |
| GCA_000021625.1 | *Vibrio cholerae* | O1 Classical Ogawa | O395 | 4,135,300 |  | 1965 |
| GCA_000006745.1 | *Vibrio cholerae* | O1 El Tor | N16961 | 4,033,464 | Bangladesh | 1975 |
| GCA_000021605.1 | *Vibrio cholerae* | O1 El Tor | M66-2 | 3,938,905 | Indonesia | 1937 |
| GCA_000022585.1 | *Vibrio cholerae* | O1 El Tor | MJ-1236 | 4,236,368 | Bangladesh | 1994 |
| GCA_000250855.1 | *Vibrio cholerae* | O1 El Tor | IEC224 | 4,079,586 | Brazil | 1990s |
| GCA_000765415.1 | *Vibrio cholerae* | O1 El Tor Ogawa | 2012EL-2176 | 4,258,023 | Haiti | 2012 |
| GCA_000829215.1 | *Vibrio cholerae* | O1 El Tor Ogawa | MS6 | 4,030,944 | Myanmar | 2008 |
| GCA_000963555.1 | *Vibrio cholerae* | O1 El Tor | FJ147 | 4,091,935 | China | 2005 |
| GCA_000969235.1 | *Vibrio cholerae* | O49 | 1154-74 | 3,928,357 | India | 1974 |
| GCA_000969265.1 | *Vibrio cholerae* | O27 | 10432-62 | 4,077,462 | Philippines | 1962 |
| GCA_001045415.1 | *Vibrio cholerae* | O1 El Tor | TSY216 | 5,000,494 | Thailand | 2010 |
| GCA_001318185.1 | *Vibrio cholerae* | O1 | KW3 | 4,089,020 |  |  |
| GCA_001887615.1 | *Vibrio cholerae* |  | NCTC 9420 | 4,076,583 | Egypt | 1954 |
| GCA_001854425.1 | *Vibrio cholerae* |  | Env-390 | 4,050,927 | Haiti | 2012 |
| GCA_001887395.1 | *Vibrio cholerae* |  | C5 | 4,102,038 | Indonesia | 1957 |
| GCA_001887415.1 | *Vibrio cholerae* |  | E1320 | 4,110,440 | China | 1974 |
| GCA_001887455.1 | *Vibrio cholerae* |  | CRC1106 | 4,099,119 | India | 1962 |

**Table S1.** Continued.

| **NCBI accession** | **Taxon name** | **Serotype** | **Strain name** | **Genome size (bp)** | **Country** | **Year** |
| --- | --- | --- | --- | --- | --- | --- |
| GCA_001887495.1 | *Vibrio cholerae* |  | E1162 | 4,110,872 | China | 1962 |
| GCA_001887635.1 | *Vibrio cholerae* |  | M2140 | 4,014,863 | Australia | 1977 |
| GCA_001887655.1 | *Vibrio cholerae* |  | E9120 | 4,066,727 | Indonesia | 1961 |
| GCA_001887475.1 | *Vibrio cholerae* |  | E506 | 4,062,508 | United States | 1974 |
| GCA_002216685.1 | *Vibrio cholerae* |  | 2521-89 | 4,123,863 | United States | 1989 |
| GCA_002313005.1 | *Vibrio cholerae* |  | ICDC-VC661 | 4,264,715 | China | 2006 |
| GCA_002313025.1 | *Vibrio cholerae* |  | FORC_055 | 4,095,733 | South Korea | 2014 |
| GCA_002073335.2 | *Vibrio cholerae* |  | FDAARGOS_223 | 4,042,269 | United States |  |
| GCA_002749635.1 | *Vibrio cholerae* |  | E7946 | 4,063,073 | Bahrain | 1978 |
| GCA_002892855.1 | *Vibrio cholerae* |  | A1552 | 4,085,463 | United States | 1992 |
| GCA_002946655.1 | *Vibrio cholerae* | O1 biovar El Tor | HC1037 | 4,061,419 | Haiti | 2014 |
| GCA_002997215.1 | *Vibrio cholerae* |  | A1552 | 4,085,460 | Peru | 1992 |
| GCA_003063785.1 | *Vibrio cholerae* |  | N16961 | 4,047,835 | Bangladesh | 1975 |
| GCA_900324455.1 | *Vibrio cholerae* |  | 4295STDY6534248 | 4,092,644 |  |  |

**Table S2.** List of genomes that was used to generate *Vibrio cholerae* species tree

| **Accession** | **Strain name** | **Genome size (bp)** | **Accession** | **Strain name** | | **Genome size (bp)** | |
| --- | --- | --- | --- | --- | --- | --- | --- |
| GCA_000195065.1 | LMA3984-4 | 3,738,715 | GCA_000961975.1 | | D-35 | | 4,010,503 |
| GCA_000021625.1 | O395 | 4,135,300 | GCA_000818865.1 | | I-1471 | | 4,034,973 |
| GCA_000152425.1 | MO10 | 4,079,638 | GCA_000829215.1 | | MS6 | | 4,030,944 |
| GCA_000021605.1 | M66-2 | 3,938,905 | GCA_000965285.1 | | R17644 | | 4,020,247 |
| GCA_000022585.1 | MJ-1236 | 4,236,368 | GCA_000966385.1 | | M818 | | 3,982,448 |
| GCA_000166455.2 | 2010EL-1786 | 4,077,740 | GCA_000966395.1 | | P18899 | | 4,015,853 |
| GCA_000250855.1 | IEC224 | 4,079,586 | GCA_000963555.1 | | FJ147 | | 4,091,935 |
| GCA_000275645.1 | H1 | 4,089,020 | GCA_000967785.1 | | I-1300 | | 4,033,785 |
| GCA_000338075.1 | G4222 | 4,202,811 | GCA_000969235.1 | | 1154-74 | | 3,928,357 |
| GCA_000953755.1 | InDRE 4354 | 4,019,937 | GCA_000969265.1 | | 10432-62 | | 4,077,462 |
| GCA_000953775.1 | InDRE 4262 | 4,019,893 | GCA_000709105.1 | | M29 | | 3,951,052 |
| GCA_000387585.1 | CP1110 | 3,925,419 | GCA_000220725.2 | | HC-49A2 | | 4,059,369 |
| GCA_000387605.1 | CP1115 | 3,927,074 | GCA_000220785.2 | | HE48 | | 4,179,415 |
| GCA_000387625.1 | CP1111 | 3,927,492 | GCA_000710155.1 | | 2012EL-1759 | | 3,982,946 |
| GCA_000387645.1 | CP1112 | 3,927,706 | GCA_000710445.1 | | RND19188 | | 3,999,500 |
| GCA_000387665.1 | CP1113 | 3,925,787 | GCA_000710455.1 | | RND19191 | | 4,005,480 |
| GCA_000387685.1 | CP1114 | 3,925,539 | GCA_000234375.2 | | HC-06A1 | | 4,028,197 |
| GCA_000387705.1 | CP1117 | 3,925,548 | GCA_000234395.2 | | HC-23A1 | | 4,099,188 |
| GCA_000387725.1 | CP1116 | 3,932,707 | GCA_000234415.2 | | HC-28A1 | | 4,027,641 |
| GCA_000765415.1 | 2012EL-2176 | 4,258,023 | GCA_000234435.2 | | HC-43A1 | | 4,053,234 |

**Table S2.** Continued.

| **Accession** | **Strain name** | **Genome size (bp)** | **Accession** | **Strain name** | | **Genome size (bp)** | |
| --- | --- | --- | --- | --- | --- | --- | --- |
| GCA_000304755.1 | CP1033(6) | 3,988,132 | GCA_001030015.1 | | YN97083 | | 4,074,242 |
| GCA_000304775.1 | HC-1A2 | 3,965,428 | GCA_001030035.1 | | YN89004 | | 3,950,281 |
| GCA_000304795.1 | HC-61A2 | 4,000,124 | GCA_000221345.1 | | HC-40A1 | | 4,031,783 |
| GCA_000305605.1 | HC-46B1 | 3,941,918 | GCA_000221365.1 | | HC-48A1 | | 4,032,180 |
| GCA_000600255.1 | L-3226 | 3,991,045 | GCA_000221385.1 | | HC-70A1 | | 4,057,434 |
| GCA_000279305.1 | CP1032(5) | 3,971,467 | GCA_000221405.1 | | HE-09 | | 3,826,131 |
| GCA_000279325.1 | CP1038(11) | 4,058,268 | GCA_000221425.1 | | HFU-02 | | 4,032,845 |
| GCA_000279245.1 | CP1041(14) | 4,085,554 | GCA_000221465.1 | | BJG-01 | | 3,910,867 |
| GCA_000279345.1 | CP1042(15) | 4,045,377 | GCA_000221485.1 | | HC-38A1 | | 4,036,059 |
| GCA_000281655.1 | CP1046(19) | 4,093,497 | GCA_000234965.1 | | HC-19A1 | | 4,033,401 |
| GCA_000279395.1 | CP1048(21) | 4,091,990 | GCA_000234945.1 | | HC-21A1 | | 4,030,433 |
| GCA_000279415.1 | HC-20A2 | 4,084,542 | GCA_000234925.1 | | HC-22A1 | | 4,034,031 |
| GCA_000279435.1 | HC-43B1 | 3,931,831 | GCA_000234905.1 | | HC-32A1 | | 4,033,209 |
| GCA_000279455.1 | HC-46A1 | 4,120,976 | GCA_000234885.1 | | HC-33A2 | | 4,024,727 |
| GCA_000279265.1 | HE-25 | 4,092,530 | GCA_000234865.1 | | HC-48B2 | | 4,024,820 |
| GCA_000279285.1 | HE-45 | 4,125,202 | GCA_000304995.1 | | HC-50A2 | | 4,030,802 |
| GCA_001027485.1 | PhVC-326 | 3,932,194 | GCA_000621645.1 | | ATCC 14035 | | 4,024,517 |
| GCA_001027495.1 | PhVE-5 | 3,929,317 | GCA_000279555.1 | | CP1030(3) | | 4,010,577 |
| GCA_001027505.1 | PhVC-311 | 3,927,966 | GCA_000302965.1 | | CP1037(10) | | 3,922,570 |
| GCA_001029975.1 | YN2011004 | 4,057,079 | GCA_000302985.1 | | CP1040(13) | | 4,033,944 |

**Table S2.** Continued.

| **Accession** | **Strain name** | **Genome size (bp)** | **Accession** | **Strain name** | | **Genome size (bp)** | |
| --- | --- | --- | --- | --- | --- | --- | --- |
| GCA_000303045.1 | CP1044(17) | 4,010,199 | GCA_000736785.1 | | 1421-77 | | 3,969,137 |
| GCA_000279785.1 | CP1047(20) | 4,039,784 | GCA_000736795.1 | | 5473-62 | | 3,913,714 |
| GCA_000303065.1 | CP1050(23) | 4,024,004 | GCA_000736845.1 | | 63-93 (MO45) | | 4,018,178 |
| GCA_000735705.1 | I-1263 | 3,957,767 | GCA_000736855.1 | | 1311-69 | | 3,973,555 |
| GCA_000302775.1 | HC-39A1 | 4,022,430 | GCA_000736865.1 | | NIH41 | | 3,952,728 |
| GCA_000302755.1 | HC-41A1 | 4,027,832 | GCA_000736875.1 | | 1157-74 | | 4,012,216 |
| GCA_000279185.1 | HC-42A1 | 4,024,780 | GCA_000736925.1 | | 981-75 | | 4,034,493 |
| GCA_000279955.1 | HC-47A1 | 4,031,718 | GCA_000736935.1 | | Aug-76 | | 4,074,796 |
| GCA_000302835.1 | HC-50A1 | 3,939,222 | GCA_000736945.1 | | 571-88 | | 4,291,048 |
| GCA_000303105.1 | HC-51A1 | 3,929,843 | GCA_000737005.1 | | 234-93 | | 4,033,463 |
| GCA_000302855.1 | HC-52A1 | 3,937,542 | GCA_000737015.1 | | 490-93 | | 3,913,946 |
| GCA_000302875.1 | HC-55A1 | 3,932,130 | GCA_000737025.1 | | 254-93 | | 3,954,364 |
| GCA_000302895.1 | HC-56A1 | 3,934,171 | GCA_000327105.3 | | HC-64A1 | | 4,032,042 |
| GCA_000279205.1 | HC-56A2 | 4,029,365 | GCA_000327125.3 | | HC-65A1 | | 4,028,731 |
| GCA_000303005.1 | HC-57A1 | 3,934,427 | GCA_000327145.3 | | HC-67A1 | | 4,026,751 |
| GCA_000279375.1 | HC-57A2 | 4,025,386 | GCA_000327165.3 | | HC-68A1 | | 4,026,495 |
| GCA_000303125.1 | HC-81A2 | 4,028,931 | GCA_000327185.3 | | HC-71A1 | | 4,028,948 |
| GCA_000303085.1 | HE-16 | 3,911,386 | GCA_000327205.3 | | HC-72A2 | | 4,028,422 |
| GCA_000736765.1 | 133-73 | 3,866,097 | GCA_000327225.3 | | HC-78A1 | | 3,937,766 |
| GCA_000736775.1 | 984-81 | 3,946,950 | GCA_000318485.2 | | HC-7A1 | | 4,074,920 |

**Table S2.** Continued.

| **Accession** | **Strain name** | **Genome size (bp)** | **Accession** | **Strain name** | | **Genome size (bp)** | |
| --- | --- | --- | --- | --- | --- | --- | --- |
| GCA_000327245.3 | HC-80A1 | 4,032,382 | GCA_000237525.2 | | 2010EL-1961 | | 4,004,160 |
| GCA_000318505.2 | HC-81A1 | 4,084,020 | GCA_000237545.2 | | 2010EL-2010H | | 4,018,270 |
| GCA_000318075.1 | PS15 | 3,910,387 | GCA_000237565.2 | | 2010EL-2010N | | 4,019,650 |
| GCA_000474965.1 | HC-36A1 | 3,959,428 | GCA_000237585.2 | | 2010V-1014 | | 4,015,902 |
| GCA_000753725.1 | MAK 676 | 3,937,648 | GCA_000237605.2 | | 2011EL-1089 | | 4,013,698 |
| GCA_000754625.1 | May-66 | 4,014,060 | GCA_000237645.2 | | 2011EL-1137 | | 4,018,022 |
| GCA_000330905.1 | 4260B | 4,039,222 | GCA_000237665.2 | | 2011V-1021 | | 4,011,850 |
| GCA_000705295.1 | M-1293 | 4,104,797 | GCA_000237685.2 | | 3500-05 | | 4,012,828 |
| GCA_000438805.2 | VCC19 | 4,134,889 | GCA_000237705.2 | | 3546-06 | | 4,012,129 |
| GCA_000763075.1 | RND81 | 3,956,193 | GCA_000237725.2 | | 3554-08 | | 4,019,938 |
| GCA_000166475.2 | 2010EL-1798 | 4,016,678 | GCA_000237765.2 | | 3582-05 | | 3,975,631 |
| GCA_000166495.2 | 2010EL-1792 | 4,014,995 | GCA_000786345.1 | | 3265/80 | | 4,012,518 |
| GCA_000223095.2 | Amazonia | 3,925,563 | GCA_000786335.1 | | 81 | | 4,011,922 |
| GCA_000740515.2 | InDRE 3140 | 4,017,985 | GCA_000257415.2 | | 2011EL-301 | | 4,002,872 |
| GCA_000237405.2 | 2009V-1046 | 4,014,220 | GCA_000939665.1 | | MAK 97 | | 3,894,875 |
| GCA_000237425.2 | 2009V-1085 | 4,014,382 | GCA_000966375.1 | | P18899-D | | 3,996,106 |
| GCA_000237445.2 | 2009V-1096 | 4,016,479 | GCA_000348365.1 | | EM-1727 | | 4,067,194 |
| GCA_000237465.2 | 2009V-1116 | 4,013,388 | GCA_000487955.1 | | E306 | | 4,165,066 |
| GCA_000237485.2 | 2009V-1131 | 4,010,678 | GCA_000788415.1 | | 2010AA-143 | | 4,027,992 |
| GCA_000237505.2 | 2010EL-1749 | 4,009,493 | GCA_000788425.1 | | 2010AA-142 | | 4,026,654 |

**Table S2.** Continued.

| **Accession** | **Strain name** | **Genome size (bp)** | **Accession** | **Strain name** | | **Genome size (bp)** | |
| --- | --- | --- | --- | --- | --- | --- | --- |
| GCA_000788435.1 | 2010AA-144 | 4,026,834 | GCA_000788915.1 | | 2012HC-17 | | 4,012,003 |
| GCA_000788495.1 | 2012Env-2 | 3,997,495 | GCA_000788935.1 | | 2012HC-32 | | 4,014,797 |
| GCA_000788535.1 | 2010AA-145 | 4,017,309 | GCA_000788955.1 | | 2012HC-35 | | 4,012,010 |
| GCA_000788555.1 | 2010AA-146 | 4,028,110 | GCA_000788975.1 | | 2012HC-33 | | 4,014,752 |
| GCA_000788575.1 | 2010AA-147 | 4,027,597 | GCA_000788995.1 | | 2012HC-21 | | 4,026,556 |
| GCA_000788595.1 | 2010AA-148 | 4,027,400 | GCA_000789015.1 | | 2012HC-22 | | 4,015,520 |
| GCA_000788615.1 | 2010AA-150 | 4,025,245 | GCA_000789035.1 | | 2012HC-11 | | 4,029,722 |
| GCA_000788635.1 | 2010AA-151 | 4,028,131 | GCA_000789055.1 | | 2012HC-16 | | 4,058,831 |
| GCA_000788655.1 | 2012Env-131 | 4,028,252 | GCA_000789075.1 | | 2012HC-15 | | 4,048,930 |
| GCA_000788675.1 | 2012Env-32 | 3,966,360 | GCA_000789095.1 | | 2012HC-19 | | 4,010,541 |
| GCA_000788695.1 | 2012Env-326 | 4,060,530 | GCA_000789115.1 | | 2012HC-08 | | 4,078,954 |
| GCA_000788735.1 | 2012Env-90 | 4,012,614 | GCA_000789135.1 | | 2012HC-10 | | 4,012,032 |
| GCA_000788755.1 | 2012Env-92 | 3,893,268 | GCA_000789155.1 | | 2012HC-12 | | 4,022,401 |
| GCA_000788775.1 | 2012HC-25 | 3,965,905 | GCA_000153865.1 | | MAK 757 | | 3,936,003 |
| GCA_000788795.1 | 2012HC-24 | 4,017,899 | GCA_000500675.1 | | RND19187 | | 4,001,659 |
| GCA_000788815.1 | 2012HC-34 | 4,014,093 | GCA_000500695.1 | | RND18899 | | 3,991,231 |
| GCA_000788835.1 | 2012HC-31 | 4,012,329 | GCA_000500715.1 | | RND6878 | | 4,000,976 |
| GCA_000788855.1 | 2012Env-94 | 4,014,775 | GCA_000500735.1 | | RND18826 | | 4,036,141 |
| GCA_000788875.1 | 2012HC-07 | 4,080,364 | GCA_000174235.1 | | VL426 | | 3,987,383 |
| GCA_000788895.1 | 2012HC-18 | 4,024,111 | GCA_000174295.1 | | TMA 21 | | 4,023,772 |

**Table S2.** Continued.

| **Accession** | **Strain name** | **Genome size (bp)** | **Accession** | **Strain name** | | **Genome size (bp)** | |
| --- | --- | --- | --- | --- | --- | --- | --- |
| GCA_000174275.1 | RC9 | 4,211,011 | GCA_001250455.1 | | A32 | | 4,002,262 |
| GCA_000174315.1 | B33 | 4,154,698 | GCA_001252875.1 | | A152 | | 3,985,453 |
| GCA_000174255.1 | TM 11079-80 | 4,055,140 | GCA_001254055.1 | | A4 | | 3,946,745 |
| GCA_000812045.1 | ZWU0020 | 4,038,248 | GCA_001254335.1 | | 4784 | | 4,018,257 |
| GCA_001045415.1 | TSY216 | 5,000,494 | GCA_001247245.1 | | 4679 | | 4,012,494 |
| GCA_000174115.1 | 12129(1) | 3,969,506 | GCA_001248505.1 | | May-12 | | 4,012,798 |
| GCA_000174335.1 | BX 330286 | 4,000,672 | GCA_001256355.1 | | PRL5 | | 3,983,009 |
| GCA_000175695.1 | CIRS 101 | 4,059,686 | GCA_001256675.1 | | A241 | | 4,021,050 |
| GCA_001248645.1 | 6214 | 4,031,671 | GCA_001257035.1 | | 4110 | | 3,918,727 |
| GCA_001250035.1 | GP145 | 3,946,053 | GCA_001257215.1 | | A330 | | 4,039,116 |
| GCA_001250435.1 | 4642 | 3,996,103 | GCA_001257975.1 | | A383 | | 3,991,423 |
| GCA_001251435.1 | 7687 | 4,030,860 | GCA_001258535.1 | | 7686 | | 4,027,393 |
| GCA_001251495.1 | GP16 | 3,968,603 | GCA_001258995.1 | | 4646 | | 4,045,251 |
| GCA_001251935.1 | 6194 | 4,022,708 | GCA_001259495.1 | | A76 | | 4,016,974 |
| GCA_001252055.1 | 4551 | 4,032,044 | GCA_001259715.1 | | 4538 | | 4,022,271 |
| GCA_001252075.1 | 4121 | 3,948,118 | GCA_001259795.1 | | A389 | | 4,094,216 |
| GCA_001252675.1 | V5 | 3,989,146 | GCA_001259995.1 | | A215 | | 3,936,701 |
| GCA_001254095.1 | A325 | 3,966,042 | GCA_001260075.1 | | GP143 | | 3,988,411 |
| GCA_001254535.1 | A59 | 3,990,340 | GCA_001261135.1 | | A245 | | 3,982,535 |
| GCA_001247885.1 | IDHO1_726 | 4,015,875 | GCA_000152465.2 | | V51 | | 4,208,620 |

**Table S2.** Continued.

| **Accession** | **Strain name** | **Genome size (bp)** | **Accession** | **Strain name** | | **Genome size (bp)** | |
| --- | --- | --- | --- | --- | --- | --- | --- |
| GCA_001249315.1 | 4322 | 3,981,548 | GCA_001184775.1 | | YN98296 | | 4,004,435 |
| GCA_001251975.1 | 6212 | 4,040,996 | GCA_001186485.1 | | EM-1626 | | 4,058,026 |
| GCA_001248465.1 | V212-1 | 3,945,950 | GCA_001186495.1 | | NHCC-078 | | 4,032,908 |
| GCA_001256015.1 | 4663 | 4,010,680 | GCA_001186505.1 | | EM-1652A | | 4,052,192 |
| GCA_001257075.1 | A488(1) | 4,022,752 | GCA_001186515.1 | | EM-1543 | | 4,064,052 |
| GCA_001257255.1 | V109 | 3,995,420 | GCA_001186565.1 | | EM-1688 | | 4,054,925 |
| GCA_001257835.1 | 6193 | 4,032,637 | GCA_001186575.1 | | EM-1654 | | 4,053,299 |
| GCA_001257895.1 | Jul-12 | 4,031,342 | GCA_001186585.1 | | EM-1690A | | 3,961,642 |
| GCA_001259055.1 | 4113 | 3,925,537 | GCA_001186595.1 | | EM-1690 | | 4,056,072 |
| GCA_001259315.1 | A131 | 4,039,161 | GCA_001186645.1 | | EM-1706 | | 3,944,903 |
| GCA_001259475.1 | 6210 | 4,035,607 | GCA_001186655.1 | | NHCC-011 | | 4,034,922 |
| GCA_001259635.1 | A68 | 3,977,514 | GCA_001253055.1 | | 4600 | | 4,028,076 |
| GCA_001259875.1 | 4661 | 3,922,782 | GCA_001258495.1 | | 4656 | | 4,035,639 |
| GCA_001260175.1 | 4662 | 4,012,468 | GCA_001258555.1 | | 4488 | | 4,012,653 |
| GCA_001260295.1 | 1362 | 4,064,557 | GCA_001259135.1 | | 4605 | | 4,018,974 |
| GCA_001260335.1 | 4339 | 4,019,959 | GCA_001259235.1 | | 4552 | | 4,033,126 |
| GCA_001260995.1 | A27 | 3,996,840 | GCA_001259555.1 | | A46 | | 4,007,597 |
| GCA_001261515.1 | A201 | 3,979,384 | GCA_001260915.1 | | A66 | | 4,012,864 |
| GCA_001261535.1 | A487(1) | 4,018,875 | GCA_001261075.1 | | PRL64 | | 4,027,134 |
| GCA_001261555.1 | 6201 | 4,022,982 | GCA_001261335.1 | | GP60 | | 3,871,365 |

**Table S2.** Continued.

| **Accession** | **Strain name** | **Genome size (bp)** | **Accession** | **Strain name** | | **Genome size (bp)** | |
| --- | --- | --- | --- | --- | --- | --- | --- |
| GCA_001186665.1 | NHCC-04 | 4,039,258 | GCA_001187085.1 | | NHCM-043 | | 4,054,814 |
| GCA_001186675.1 | NHCC-048 | 4,041,973 | GCA_001187095.1 | | NHCM-045 | | 4,036,868 |
| GCA_001186725.1 | NHCC-021 | 4,044,257 | GCA_001187105.1 | | NHCM-053 | | 4,056,015 |
| GCA_001186735.1 | NHCC-042 | 4,040,251 | GCA_001187145.1 | | NHCM-047 | | 4,041,405 |
| GCA_001186755.1 | NHCC-019 | 4,006,456 | GCA_001187165.1 | | NHCM-054 | | 4,037,409 |
| GCA_001186785.1 | NHCC-05 | 4,063,046 | GCA_001187175.1 | | NHCM-048 | | 3,956,153 |
| GCA_001186805.1 | NHCC-081 | 4,036,135 | GCA_001187185.1 | | NHCC-068 | | 4,033,814 |
| GCA_001186825.1 | NHCM-01 | 4,058,157 | GCA_001187225.1 | | NHCC-079 | | 4,037,755 |
| GCA_001186835.1 | NHCC-083 | 4,032,941 | GCA_001187245.1 | | NHCC-080 | | 4,032,145 |
| GCA_001186855.1 | NHCM-02 | 4,058,043 | GCA_001187255.1 | | EM-1542 | | 4,055,638 |
| GCA_001186885.1 | NHCM-06 | 4,065,293 | GCA_001187265.1 | | NHCM-03 | | 4,061,995 |
| GCA_001186905.1 | NHCM-04 | 4,051,907 | GCA_001254895.1 | | MG116025 | | 4,027,317 |
| GCA_001186915.1 | NHCM-012 | 4,058,997 | GCA_001248865.1 | | A193 | | 4,006,062 |
| GCA_001186925.1 | NHCM-013 | 4,056,172 | GCA_001248905.1 | | A70 | | 3,981,994 |
| GCA_001186965.1 | NHCM-016A | 4,054,882 | GCA_001248945.1 | | A213 | | 3,943,920 |
| GCA_001186985.1 | NHCM-017 | 4,270,351 | GCA_001249085.1 | | MBRN14 | | 4,014,074 |
| GCA_001186995.1 | NHCM-029 | 4,058,323 | GCA_001249515.1 | | 6191 | | 4,031,393 |
| GCA_001187015.1 | NHCM-044 | 4,031,872 | GCA_001249715.1 | | GP152 | | 3,964,217 |
| GCA_001187025.1 | NHCM-037 | 4,053,555 | GCA_001249795.1 | | 4672 | | 3,972,315 |
| GCA_001187065.1 | NHCM-033 | 4,091,543 | GCA_001249995.1 | | A177 | | 4,009,459 |

**Table S2.** Continued.

| **Accession** | **Strain name** | **Genome size (bp)** | **Accession** | **Strain name** | | **Genome size (bp)** | |
| --- | --- | --- | --- | --- | --- | --- | --- |
| GCA_001250195.1 | MJ1485 | 4,112,661 | GCA_001254635.1 | | 4623 | | 4,034,153 |
| GCA_001250615.1 | A488(2) | 4,017,885 | GCA_001254655.1 | | A10 | | 4,145,031 |
| GCA_001250795.1 | MBN17 | 4,019,692 | GCA_001254675.1 | | A5 | | 4,032,311 |
| GCA_001250935.1 | A61 | 3,966,297 | GCA_001254815.1 | | 4675 | | 3,985,607 |
| GCA_001252495.1 | A18 | 4,012,358 | GCA_001254955.1 | | 6197 | | 4,030,166 |
| GCA_001252775.1 | Jan-17 | 4,033,005 | GCA_001255155.1 | | A22 | | 3,979,233 |
| GCA_001252855.1 | 4111 | 3,870,008 | GCA_001255295.1 | | A200 | | 4,005,389 |
| GCA_001252895.1 | Jul-12 | 4,028,120 | GCA_001255575.1 | | A6 | | 3,998,598 |
| GCA_001253035.1 | 1346 | 4,069,009 | GCA_001255835.1 | | 4536 | | 4,024,336 |
| GCA_001253155.1 | A154 | 3,982,016 | GCA_001255915.1 | | 7685 | | 4,024,204 |
| GCA_001253235.1 | A29 | 4,000,671 | GCA_001247525.1 | | A346(1) | | 4,095,237 |
| GCA_001253295.1 | A185 | 4,009,761 | GCA_001247835.1 | | 1627 | | 4,065,829 |
| GCA_001253315.1 | GP140 | 3,919,716 | GCA_001248135.1 | | A186 | | 4,004,225 |
| GCA_001253455.1 | 4122 | 3,747,369 | GCA_001248195.1 | | A60 | | 3,997,697 |
| GCA_001253575.1 | GP8 | 3,951,857 | GCA_000153785.3 | | AM-19226 | | 4,053,126 |
| GCA_001253695.1 | A31 | 4,002,336 | GCA_000153985.3 | | MZO-2 | | 3,977,246 |
| GCA_001253835.1 | A49 | 4,003,129 | GCA_000154005.2 | | 623-39 | | 4,164,181 |
| GCA_001254355.1 | MG116226 | 4,028,960 | GCA_001597715.1 | | I-1181 | | 2,920,335 |
| GCA_001254435.1 | GP160 | 3,986,399 | GCA_001617665.1 | | CW-6 | | 3,921,611 |
| GCA_001254575.1 | A103 | 4,019,492 | GCA_001617675.1 | | M888D | | 3,970,532 |

**Table S2.** Continued.

| **Accession** | **Strain name** | **Genome size (bp)** | **Accession** | **Strain name** | | **Genome size (bp)** | |
| --- | --- | --- | --- | --- | --- | --- | --- |
| GCA_000167935.2 | V52 | 4,045,303 | GCA_000305135.2 | | HE-46 | | 3,874,516 |
| GCA_000168895.2 | 1587 | 4,216,194 | GCA_000305195.2 | | HC-59A1 | | 3,934,654 |
| GCA_000168935.3 | MZO-3 | 4,137,330 | GCA_000305525.2 | | HC-02C1 | | 3,935,576 |
| GCA_000220745.3 | HCUF01 | 4,067,550 | GCA_000305545.2 | | HC-59B1 | | 3,934,504 |
| GCA_000220765.3 | HE39 | 3,937,798 | GCA_000305565.2 | | HC-44C1 | | 3,876,537 |
| GCA_000234455.3 | HC-61A1 | 4,067,936 | GCA_000305585.2 | | HC-37A1 | | 4,029,550 |
| GCA_000237745.2 | 3569-08 | 3,958,670 | GCA_000305625.2 | | HC-62B1 | | 4,028,528 |
| GCA_000299495.2 | VC35 | 3,912,714 | GCA_000305675.2 | | HC-17A2 | | 4,023,476 |
| GCA_000299515.2 | VC1761 | 4,011,457 | GCA_000305695.2 | | HC-69A1 | | 4,024,183 |
| GCA_000299535.2 | VC4370 | 3,986,677 | GCA_001402255.1 | | YB4G06 | | 4,032,120 |
| GCA_000569115.2 | PCS-022 | 4,055,383 | GCA_001402185.1 | | YB1A01 | | 3,876,619 |
| GCA_000304915.2 | CP1035(8) | 3,933,356 | GCA_001402265.1 | | YB4F05 | | 3,886,413 |
| GCA_000304935.2 | HC-17A1 | 4,174,355 | GCA_001402275.1 | | YB3G04 | | 4,031,104 |
| GCA_000304955.2 | HC-41B1 | 3,883,402 | GCA_001402335.1 | | YB7A06 | | 3,881,934 |
| GCA_000305015.2 | HC-55C2 | 3,940,186 | GCA_001402285.1 | | YB4C07 | | 4,015,430 |
| GCA_000305055.2 | HC-60A1 | 3,938,002 | GCA_001402365.1 | | YB1G06 | | 3,938,799 |
| GCA_000305075.2 | HC-62A1 | 4,032,880 | GCA_001402375.1 | | YB2A06 | | 4,033,481 |
| GCA_000305095.2 | HC-77A1 | 4,040,127 | GCA_001402415.1 | | YB2G05 | | 3,888,413 |
| GCA_000305115.2 | HE-40 | 3,875,898 | GCA_001402425.1 | | YB2G07 | | 3,941,216 |
| GCA_000305645.2 | HC-55B2 | 3,934,012 | GCA_001402435.1 | | YB5A06 | | 3,886,853 |

**Table S2.** Continued.

| **Accession** | **Strain name** | **Genome size (bp)** | **Accession** | **Strain name** | | **Genome size (bp)** | |
| --- | --- | --- | --- | --- | --- | --- | --- |
| GCA_001402445.1 | YB6A06 | 3,884,851 | GCA_000348225.2 | | EC-0051 | | 4,069,374 |
| GCA_001402535.1 | YB2A05 | 3,886,789 | GCA_000348245.2 | | EDC-020 | | 4,028,191 |
| GCA_001402545.1 | YB3B05 | 4,014,368 | GCA_000348265.2 | | EDC-022 | | 4,050,458 |
| GCA_001402575.1 | YB4G05 | 3,927,241 | GCA_000348305.2 | | EM-1546 | | 4,061,605 |
| GCA_001402585.1 | YB4H02 | 4,039,090 | GCA_000348285.2 | | EM-1536 | | 4,053,577 |
| GCA_001402595.1 | YB7A09 | 3,885,495 | GCA_000348345.2 | | EM-1676A | | 4,007,225 |
| GCA_001402605.1 | YB4B03 | 3,916,598 | GCA_000348385.2 | | NHCC-004A | | 4,031,643 |
| GCA_001402655.1 | YB8E08 | 4,013,838 | GCA_000348405.2 | | NHCC-006C | | 4,028,239 |
| GCA_001402745.1 | 877-163 | 3,864,837 | GCA_000348425.2 | | NHCC-008D | | 3,971,576 |
| GCA_001411585.1 | YB2G01 | 4,032,885 | GCA_000348445.2 | | NHCC-010F | | 4,028,811 |
| GCA_000338215.2 | P-18785 | 3,978,895 | GCA_000348465.2 | | Nep-21106 | | 4,031,227 |
| GCA_000348045.2 | 116059 | 4,017,055 | GCA_000348485.2 | | Nep-21113 | | 4,042,420 |
| GCA_000348065.2 | 116063 | 3,970,379 | GCA_000348505.2 | | PCS-023 | | 4,031,594 |
| GCA_000348085.2 | 87395 | 3,858,111 | GCA_001281585.1 | | 31 | | 4,041,947 |
| GCA_000348105.2 | 95412 | 4,030,154 | GCA_001281595.1 | | 39 | | 4,024,490 |
| GCA_000348125.2 | AG-7404 | 3,915,123 | GCA_001281615.1 | | 43 | | 4,181,484 |
| GCA_000348145.2 | AG-8040 | 3,977,194 | GCA_001281665.1 | | 56 | | 4,105,143 |
| GCA_000348165.2 | EC-0009 | 4,028,271 | GCA_001282605.1 | | EC-051 | | 4,068,854 |
| GCA_000348185.2 | EC-0012 | 4,031,923 | GCA_001292745.1 | | 116-17b | | 4,125,773 |
| GCA_000348205.2 | EC-0027 | 4,027,674 | GCA_001292785.1 | | BRV8 | | 4,109,032 |

**Table S2.** Continued.

| **Accession** | **Strain name** | **Genome size (bp)** | **Accession** | **Strain name** | | **Genome size (bp)** | |
| --- | --- | --- | --- | --- | --- | --- | --- |
| GCA_001515085.1 | M1399 | 4,013,390 | GCA_001887615.1 | | NCTC 9420 | | 4,076,583 |
| GCA_001515105.1 | M1395 | 4,028,990 | GCA_000788715.2 | | 2012Env-9 | | 4,061,813 |
| GCA_001515115.1 | M988 | 3,995,831 | GCA_001854425.1 | | Env-390 | | 4,050,927 |
| GCA_001517845.1 | M1275 | 4,124,381 | GCA_001887395.1 | | C5 | | 4,102,038 |
| GCA_001318185.1 | KW3 | 4,089,020 | GCA_001887415.1 | | E1320 | | 4,110,440 |
| GCA_001521835.1 | M888 | 4,041,101 | GCA_001887435.1 | | CRC711 | | 4,057,520 |
| GCA_001543465.1 | PIC018 | 4,029,307 | GCA_001887455.1 | | CRC1106 | | 4,099,119 |
| GCA_001543505.1 | Drakes2013 | 4,042,766 | GCA_001887495.1 | | E1162 | | 4,110,872 |
| GCA_001515165.1 | M1522 | 3,955,145 | GCA_001887635.1 | | M2140 | | 4,014,863 |
| GCA_001661905.1 | I-1187 | 3,957,421 | GCA_001887655.1 | | E9120 | | 4,066,727 |
| GCA_001683415.1 | 2740-80 | 4,088,961 | GCA_001887475.1 | | E506 | | 4,062,508 |
| GCA_001637545.1 | M139 | 3,972,882 | GCA_001718095.1 | | L15 | | 4,083,945 |
| GCA_001637555.1 | M299 | 3,957,801 | GCA_001729125.1 | | 857 | | 3,993,682 |
| GCA_001637575.1 | M1501 | 4,011,026 | GCA_001729195.1 | | VC22 | | 4,022,142 |
| GCA_001639085.1 | P13762 | 4,096,073 | GCA_001743085.1 | | 3223-74 | | 4,017,920 |
| GCA_001641685.1 | M1518 | 4,014,173 | GCA_001857145.1 | | 2559-78 | | 3,982,589 |
| GCA_001641705.1 | M1524 | 3,961,165 | GCA_001857155.1 | | VC53 | | 4,217,958 |
| GCA_001641745.1 | Jun-67 | 4,006,954 | GCA_001857165.1 | | VC48 | | 3,931,795 |
| GCA_001641765.1 | M1327 | 4,119,805 | GCA_001857175.1 | | VC56 | | 4,193,045 |
| GCA_001887515.1 | NCTC 5395 | 4,170,245 | GCA_001857225.1 | | 2631-78 | | 3,943,436 |

**Table S2.** Continued.

| **Accession** | **Strain name** | **Genome size (bp)** | **Accession** | **Strain name** | | **Genome size (bp)** | |
| --- | --- | --- | --- | --- | --- | --- | --- |
| GCA_001857245.1 | 2512-86 | 3,999,880 | GCA_001860225.1 | | CMR001 | | 4,022,419 |
| GCA_001857265.1 | 3272-78 | 3,946,798 | GCA_001858585.1 | | CMR004 | | 4,021,213 |
| GCA_001857285.1 | 692-79 | 3,938,077 | GCA_001860265.1 | | CMR007 | | 4,026,765 |
| GCA_001857305.1 | 2479-86 | 4,011,444 | GCA_001860285.1 | | CMR008 | | 4,032,975 |
| GCA_001857325.1 | 1496-86 | 3,972,280 | GCA_001860295.1 | | CMR009 | | 4,029,303 |
| GCA_001857345.1 | 2523-87 | 3,947,558 | GCA_001860315.1 | | CMR010 | | 4,035,994 |
| GCA_001857365.1 | 3225-74 | 4,002,038 | GCA_001860345.1 | | CMR011 | | 4,024,152 |
| GCA_001857425.1 | 2633-78 | 3,902,363 | GCA_001860365.1 | | CMR012 | | 4,027,803 |
| GCA_001857405.1 | 1074-78 | 3,967,821 | GCA_001860385.1 | | CMR013 | | 4,034,667 |
| GCA_001857435.1 | C6706 | 4,019,194 | GCA_001860395.1 | | CMR014 | | 4,042,618 |
| GCA_001857455.1 | SIO | 3,998,588 | GCA_001860425.1 | | CMR015 | | 4,027,621 |
| GCA_001857485.1 | TP | 4,060,799 | GCA_001860445.1 | | CMR016 | | 4,026,175 |
| GCA_001857505.1 | 3568-07 | 4,056,633 | GCA_001860465.1 | | CMR017 | | 4,034,135 |
| GCA_001857515.1 | HE46 | 3,978,873 | GCA_001860485.1 | | CMR018 | | 4,036,389 |
| GCA_001718105.1 | L11 | 4,000,957 | GCA_001899465.1 | | 76 | | 4,000,689 |
| GCA_001735565.1 | S12 | 4,061,577 | GCA_001953365.1 | | DL4211 | | 3,985,387 |
| GCA_001858445.1 | CMR020 | 4,034,563 | GCA_001953375.1 | | DL4215 | | 3,981,208 |
| GCA_001858455.1 | CMR021 | 4,037,271 | GCA_002078825.1 | | Mar-48 | | 4,045,510 |
| GCA_001858465.1 | CMR022 | 4,031,968 | GCA_002078815.1 | | 20390 | | 4,045,740 |
| GCA_001858475.1 | CMR019 | 4,028,526 | GCA_002078635.1 | | 39361 | | 4,024,330 |

**Table S2.** Continued.

| **Accession** | **Strain name** | **Genome size (bp)** | **Accession** | **Strain name** | | **Genome size (bp)** | |
| --- | --- | --- | --- | --- | --- | --- | --- |
| GCA_002078755.1 | TEM/29/01-003 | 4,041,320 | GCA_002097815.1 | | CISM_1163068.5 | | 4,014,933 |
| GCA_002078595.1 | 43Ki | 4,048,514 | GCA_002097825.1 | | CISM_780298.0 | | 4,194,523 |
| GCA_002078795.1 | J8YRS KAGUNGA | 4,044,226 | GCA_002097835.1 | | CISM_770180.8 | | 4,190,491 |
| GCA_002078715.1 | 19886 | 4,039,107 | GCA_002097845.1 | | CISM_770067.4 | | 4,193,261 |
| GCA_002078705.1 | 21027 | 4,043,733 | GCA_002097895.1 | | CISM_710180.8 | | 4,193,899 |
| GCA_002078695.1 | TEM/15/01-005 | 4,043,231 | GCA_002097905.1 | | CISM_740115.4 | | 4,034,260 |
| GCA_002078055.1 | 36KI | 4,020,592 | GCA_002097925.1 | | CISM_655665.0 | | 4,037,096 |
| GCA_002076155.1 | O1S | 4,024,114 | GCA_002097915.1 | | CISM_769845.7 | | 4,195,639 |
| GCA_002076165.1 | O7S | 4,023,827 | GCA_002097975.1 | | CISM_655630.3 | | 4,061,319 |
| GCA_002076235.1 | TEM/25/01-004 | 4,042,340 | GCA_002097985.1 | | CISM_511 | | 4,034,679 |
| GCA_002076245.1 | O9S | 4,022,009 | GCA_002097995.1 | | CISM_655664.3 | | 4,167,720 |
| GCA_002076255.1 | O2 | 4,044,276 | GCA_002098005.1 | | CISM_510 | | 4,038,804 |
| GCA_002076455.1 | OO4 | 4,025,417 | GCA_002098055.1 | | CISM_420 | | 4,029,921 |
| GCA_002076485.1 | 47623 | 4,022,899 | GCA_002098065.1 | | CISM_505 | | 4,038,667 |
| GCA_002076645.1 | 47610 | 4,022,193 | GCA_002098075.1 | | CISM_399 | | 4,036,149 |
| GCA_002076695.1 | 8Mo | 4,023,189 | GCA_002098085.1 | | CISM_398 | | 4,079,517 |
| GCA_002097735.1 | CISM_300055 | 4,148,676 | GCA_002098135.1 | | CISM_382 | | 4,038,086 |
| GCA_002097745.1 | CISM_300205 | 3,937,137 | GCA_002098145.1 | | CISM_375 | | 4,031,104 |
| GCA_002097755.1 | CISM_300506 | 4,026,639 | GCA_002098155.1 | | CISM_374 | | 4,040,267 |
| GCA_002097765.1 | CISM_S/Nida | 4,195,572 | GCA_002098195.1 | | CISM_347 | | 4,049,584 |

**Table S2.** Continued.

| **Accession** | **Strain name** | **Genome size (bp)** | **Accession** | **Strain name** | | **Genome size (bp)** | |
| --- | --- | --- | --- | --- | --- | --- | --- |
| GCA_002098215.1 | CISM_326 | 4,163,534 | GCA_002098625.1 | | CISM_134 | | 4,194,365 |
| GCA_002098225.1 | CISM_302015 | 4,207,521 | GCA_002098605.1 | | CISM_122 | | 4,032,037 |
| GCA_002098235.1 | CISM_302029 | 4,221,472 | GCA_002098655.1 | | CISM_121 | | 4,034,073 |
| GCA_002098295.1 | CISM_300043 | 4,038,332 | GCA_002098675.1 | | CISM_120 | | 4,036,655 |
| GCA_002098255.1 | CISM_300215 | 4,065,320 | GCA_002098695.1 | | CISM_105 | | 4,195,830 |
| GCA_002098305.1 | CISM_296 | 4,030,126 | GCA_002098705.1 | | CISM_1020234.0 | | 4,037,201 |
| GCA_002098335.1 | CISM_300209 | 4,036,397 | GCA_002098715.1 | | CISM_1020231.9 | | 4,026,090 |
| GCA_002098345.1 | CISM_300208 | 4,039,049 | GCA_002098755.1 | | CISM_1020229.6 | | 4,034,980 |
| GCA_002098355.1 | CISM_196 | 4,195,851 | GCA_002098765.1 | | CISM_101 | | 4,034,875 |
| GCA_002098365.1 | CISM_191 | 4,193,176 | GCA_002098795.1 | | CISM_1019829.2 | | 4,031,863 |
| GCA_002098415.1 | CISM_188 | 4,196,807 | GCA_002098805.1 | | CISM_1019828.5 | | 4,036,202 |
| GCA_002098425.1 | CISM_189 | 4,185,376 | GCA_002098835.1 | | CISM_100 | | 4,040,221 |
| GCA_002098435.1 | CISM_179 | 4,194,264 | GCA_002098885.1 | | CISM_0091 | | 4,036,548 |
| GCA_002098445.1 | CISM_178 | 4,193,944 | GCA_002098875.1 | | CISM_0079 | | 4,040,112 |
| GCA_002098495.1 | CISM_152 | 4,035,506 | GCA_002098845.1 | | CISM_091 | | 4,162,346 |
| GCA_002098515.1 | CISM_154 | 4,032,160 | GCA_002098915.1 | | CISM_0074 | | 4,035,667 |
| GCA_002098525.1 | CISM_151 | 4,037,351 | GCA_002098935.1 | | CISM_0035 | | 4,034,640 |
| GCA_002098535.1 | CISM_153 | 4,162,416 | GCA_002098955.1 | | CISM_0034 | | 4,036,351 |
| GCA_002098555.1 | CISM_147 | 4,196,567 | GCA_002098965.1 | | CISM_0019 | | 4,033,489 |
| GCA_002098595.1 | CISM_146 | 4,194,205 | GCA_002098995.1 | | CISM_0018 | | 4,036,015 |

**Table S2.** Continued.

| **Accession** | **Strain name** | **Genome size (bp)** | **Accession** | **Strain name** | | **Genome size (bp)** | |
| --- | --- | --- | --- | --- | --- | --- | --- |
| GCA_002099015.1 | CISM_0017 | 4,036,556 | GCA_002076585.1 | | O5MU | | 4,023,442 |
| GCA_002099035.1 | CISM_0016 | 4,032,775 | GCA_002076615.1 | | 7Mo | | 4,022,979 |
| GCA_002099055.1 | CISM_0015 | 4,038,651 | GCA_002076635.1 | | 9Mo | | 4,024,326 |
| GCA_002099065.1 | CISM_0014 | 4,032,716 | GCA_002076665.1 | | 5Mo | | 4,023,405 |
| GCA_002099095.1 | CISM_0010 | 4,157,755 | GCA_002076705.1 | | 2Mo | | 4,024,773 |
| GCA_002099115.1 | CISM_0008 | 4,035,087 | GCA_002076735.1 | | TEM/12/12-001 | | 4,041,711 |
| GCA_002099125.1 | CISM_0005 | 4,034,250 | GCA_002076745.1 | | TEM/04/01-001 | | 4,042,748 |
| GCA_002102575.1 | G_33 | 3,933,642 | GCA_002076775.1 | | 21B | | 4,045,625 |
| GCA_002114205.1 | 617 | 3,960,877 | GCA_002076785.1 | | 20478 | | 4,046,809 |
| GCA_002076185.1 | 11S | 4,025,935 | GCA_002216685.1 | | 2521-89 | | 4,123,863 |
| GCA_002076175.1 | O6MU | 4,025,207 | GCA_002194155.1 | | FC1877 | | 3,984,202 |
| GCA_002076265.1 | TEM/10/01-002 | 4,044,593 | GCA_002194165.1 | | FC3611a | | 4,013,511 |
| GCA_002076315.1 | 48055 | 4,024,390 | GCA_002194185.1 | | FC3611b | | 4,013,713 |
| GCA_002076415.1 | 7714 | 4,022,767 | GCA_002194215.1 | | FC2273 | | 4,022,199 |
| GCA_002076425.1 | 1Mo | 4,024,350 | GCA_002194245.1 | | FC1384 | | 4,014,742 |
| GCA_002076465.1 | O3MU | 4,025,781 | GCA_002194235.1 | | FC2271 | | 4,016,919 |
| GCA_002076475.1 | 39Ki | 4,048,610 | GCA_002194265.1 | | FC1341 | | 3,999,105 |
| GCA_002076535.1 | 31Ki | 4,044,237 | GCA_002194295.1 | | FC1105 | | 3,960,610 |
| GCA_002076545.1 | O7MU | 4,025,010 | GCA_002194305.1 | | FC1817 | | 4,043,413 |
| GCA_002076575.1 | O3S | 4,022,861 | GCA_002194335.1 | | FC1225 | | 4,003,412 |

**Table S2.** Continued.

| **Accession** | **Strain name** | **Genome size (bp)** | **Accession** | **Strain name** | | **Genome size (bp)** | |
| --- | --- | --- | --- | --- | --- | --- | --- |
| GCA_002196055.1 | P-18748 | 3,961,379 | GCA_002251495.1 | | NMH2016 | | 3,754,606 |
| GCA_002196065.1 | P-18778 | 4,012,469 | GCA_002284495.1 | | OYP1G01 | | 3,969,671 |
| GCA_002196095.1 | 102 | 3,995,638 | GCA_002284425.1 | | OYP2D07 | | 4,082,264 |
| GCA_002196105.1 | 147 | 4,107,181 | GCA_002284475.1 | | OYP2C05 | | 3,989,036 |
| GCA_002196135.1 | 89 | 4,115,283 | GCA_002284315.1 | | OYP4H06 | | 3,907,548 |
| GCA_002196155.1 | Jun-00 | 4,117,933 | GCA_002284245.1 | | OYP4H11 | | 3,934,959 |
| GCA_002196175.1 | 28 | 4,076,049 | GCA_002284175.1 | | OYP6F08 | | 3,912,172 |
| GCA_002196165.1 | Jan-00 | 3,960,426 | GCA_002284205.1 | | OYP6D06 | | 4,036,442 |
| GCA_002196225.1 | 114 | 3,971,369 | GCA_002284155.1 | | OYP6G08 | | 3,950,949 |
| GCA_002196255.1 | 85 | 3,979,842 | GCA_002284455.1 | | OYP1E07 | | 3,942,520 |
| GCA_002196305.1 | 433 | 3,984,043 | GCA_002284415.1 | | OYP2E01 | | 3,966,741 |
| GCA_002196275.1 | M1344 | 4,029,820 | GCA_002284395.1 | | OYP2A12 | | 4,068,380 |
| GCA_002196335.1 | M1030 | 3,951,787 | GCA_002284325.1 | | OYP4G08 | | 3,927,912 |
| GCA_002196375.1 | M1337 | 4,005,292 | GCA_002284265.1 | | OYP6F10 | | 3,860,908 |
| GCA_002196395.1 | 34Kayum | 3,985,205 | GCA_002284185.1 | | OYP6E07 | | 3,957,612 |
| GCA_002204075.1 | 39 | 3,866,753 | GCA_002284125.1 | | OYP8A01 | | 3,926,089 |
| GCA_002204085.1 | 866 | 3,892,514 | GCA_002284115.1 | | OYP8F12 | | 4,038,901 |
| GCA_002204095.1 | Jun-00 | 4,017,602 | GCA_002284355.1 | | OYP3F10 | | 3,937,113 |
| GCA_002204105.1 | 56 | 3,950,861 | GCA_002284235.1 | | OYP5F10 | | 4,088,901 |
| GCA_002217575.1 | W4-13 | 3,997,832 | GCA_002284095.1 | | OYP8C06 | | 4,033,034 |

**Table S2.** Continued.

| **Accession** | **Strain name** | **Genome size (bp)** | **Accession** | **Strain name** | | **Genome size (bp)** | |
| --- | --- | --- | --- | --- | --- | --- | --- |
| GCA_002313005.1 | ICDC-VC661 | 4,264,715 | GCA_002808435.1 | | 22043204_C1 | | 4,043,072 |
| GCA_002313025.1 | FORC_055 | 4,095,733 | GCA_002808485.1 | | 330013_C1 | | 4,041,908 |
| GCA_002407455.1 | VC0101557 | 3,863,050 | GCA_002807735.1 | | S002506 | | 4,042,063 |
| GCA_001250235.2 | A19 | 4,033,501 | GCA_002807765.1 | | 330073_A | | 4,042,676 |
| GCA_001471585.2 | FDAARGOS_103 | 4,036,048 | GCA_002807785.1 | | 330113 | | 4,042,653 |
| GCA_001471455.2 | ATCC 11629 | 4,216,088 | GCA_002807825.1 | | 220076-6 | | 4,043,584 |
| GCA_001525525.2 | FDAARGOS_102 | 4,071,484 | GCA_002807835.1 | | S002300_B | | 3,949,769 |
| GCA_002073335.2 | FDAARGOS_223 | 4,042,269 | GCA_002807865.1 | | 220075-6 | | 3,954,018 |
| GCA_002196295.1 | 2403 | 4,745,993 | GCA_002807875.1 | | 330920_B | | 4,044,638 |
| GCA_002807705.1 | 330590 | 4,041,648 | GCA_002807895.1 | | 330110 | | 4,043,457 |
| GCA_002807725.1 | S002502 | 3,946,275 | GCA_002807925.1 | | S023208 | | 4,027,340 |
| GCA_002807805.1 | S003806 | 4,046,403 | GCA_002807945.1 | | 330920_A | | 4,042,529 |
| GCA_002807985.1 | S002300_E | 4,042,300 | GCA_002807975.1 | | S003202 | | 4,039,674 |
| GCA_002808075.1 | S000600_C10 | 4,041,449 | GCA_002807965.1 | | 330073_B | | 4,045,868 |
| GCA_002808215.1 | 22043300_C6 | 4,042,333 | GCA_002808065.1 | | 330440_C1 | | 4,043,032 |
| GCA_002808265.1 | 330033_C1 | 4,043,006 | GCA_002808105.1 | | 330081 | | 4,044,553 |
| GCA_002808365.1 | S000100_C5 | 4,075,565 | GCA_002808125.1 | | S023202 | | 4,042,056 |
| GCA_002808305.1 | S042408 | 3,999,339 | GCA_002808145.1 | | 22087102_C2 | | 4,042,349 |
| GCA_002808405.1 | S081300_C2 | 4,042,531 | GCA_002808155.1 | | S002604 | | 4,045,052 |
| GCA_002808415.1 | 331721_C1 | 4,042,872 | GCA_002808165.1 | | 22043202_C1 | | 4,044,047 |

**Table S2.** Continued.

| **Accession** | **Strain name** | **Genome size (bp)** | **Accession** | **Strain name** | | **Genome size (bp)** | |
| --- | --- | --- | --- | --- | --- | --- | --- |
| GCA_002808205.1 | S003008 | 4,044,346 | GCA_003057015.1 | | 8 | | 3,903,289 |
| GCA_002808225.1 | 330898_C2 | 4,041,730 | GCA_003057035.1 | | 3178 | | 3,998,812 |
| GCA_002808275.1 | 22044108_C3 | 4,042,366 | GCA_003057075.1 | | 2687 | | 3,966,695 |
| GCA_002808325.1 | S040602_C1 | 4,043,126 | GCA_003057085.1 | | 124 | | 3,969,210 |
| GCA_002808345.1 | 22087500_C9 | 4,040,182 | GCA_003057115.1 | | 2843 | | 4,001,056 |
| GCA_002808355.1 | S042100 | 4,042,390 | GCA_003057775.1 | | A3_296 | | 3,774,522 |
| GCA_002808465.1 | 22043200_C1 | 4,042,896 | GCA_003063785.1 | | N16961 | | 4,047,835 |
| GCA_002843255.1 | VcN1 | 4,145,933 | GCA_003063885.1 | | Sa5Y | | 4,050,878 |
| GCA_900185995.1 | BC1071 | 4,194,576 | GCA_003097695.1 | | A1552 | | 4,085,468 |
| GCA_002749635.1 | E7946 | 4,063,073 | GCA_900324425.1 | | 4295STDY6534232 | | 4,092,645 |
| GCA_002890525.1 | 11116 | 4,059,991 | GCA_900324445.1 | | 4295STDY6534216 | | 4,092,641 |
| GCA_002911455.1 | 5879 | 3,982,623 | GCA_900324455.1 | | 4295STDY6534248 | | 4,092,644 |
| GCA_002946655.1 | HC1037 | 4,061,419 | GCA_003130475.1 | | 41D | | 4,038,688 |
| GCA_003015005.1 | 186 | 4,019,066 | GCA_003130465.1 | | 16241D | | 4,081,404 |
| GCA_003013485.1 | 2044 | 4,045,779 | GCA_003130485.1 | | 169D | | 4,102,946 |
| GCA_003056705.1 | 20-a_11 | 4,111,774 | GCA_003130495.1 | | 1270D | | 4,016,316 |
| GCA_003056955.1 | M1425 | 4,025,851 | GCA_003096115.1 | | Ogawa 18963 | | 3,966,209 |
| GCA_003056975.1 | May-07 | 3,970,206 | GCA_003096135.1 | | Jan-26 | | 3,997,109 |
| GCA_003056995.1 | M1332 | 3,975,667 | GCA_002899735.1 | | IDH-06787 | | 4,404,109 |
| GCA_003057055.1 | 2613 | 3,967,932 | GCA_002918335.1 | | 146P | | 4,012,364 |

**Table S2.** Continued.

| **Accession** | **Strain name** | **Genome size (bp)** | **Accession** | **Strain name** | | **Genome size (bp)** | |
| --- | --- | --- | --- | --- | --- | --- | --- |
| GCA_002918345.1 | 146N | 4,014,421 | GCA_003311945.1 | | 2014V-1107 | | 3,987,160 |
| GCA_003205555.1 | UG054 | 4,041,743 | GCA_003311965.1 | | 2016V-1111 | | 3,896,089 |
| GCA_003205655.1 | UG042 | 4,044,573 | GCA_003311975.1 | | 2017V-1105 | | 4,080,506 |
| GCA_003205635.1 | UG020 | 4,023,664 | GCA_003312005.1 | | 2017V-1110 | | 3,993,568 |
| GCA_003205565.1 | UG046 | 4,042,522 | GCA_003312035.1 | | 2016V-1018 | | 4,189,996 |
| GCA_003205685.1 | UG026 | 4,024,131 | GCA_003312015.1 | | 2017V-1144 | | 3,868,307 |
| GCA_003205705.1 | UG071 | 4,034,068 | GCA_003312065.1 | | 2016V-1091 | | 3,814,012 |
| GCA_003205675.1 | UG086 | 4,035,264 | GCA_003312085.1 | | 2016V-1114 | | 3,872,198 |
| GCA_003205755.1 | UG060 | 4,036,290 | GCA_003312095.1 | | 2017V-1176 | | 3,896,121 |
| GCA_003205735.1 | UG040 | 4,036,071 |  | |  | |  |
| GCA_003205765.1 | UG010 | 4,007,874 |  | |  | |  |
| GCA_003260135.1 | 60555434 | 3,918,371 |  | |  | |  |
| GCA_003311755.1 | 2523-88 | 4,180,641 |  | |  | |  |
| GCA_003311805.1 | 2017V-1038 | 4,028,322 |  | |  | |  |
| GCA_003311815.1 | 2015V-1076 | 4,050,103 |  | |  | |  |
| GCA_003311825.1 | 2016V-1062 | 4,021,660 |  | |  | |  |
| GCA_003311885.1 | 2017V-1124 | 4,137,761 |  | |  | |  |
| GCA_003311865.1 | 2017V-1070 | 4,082,510 |  | |  | |  |
| GCA_003311895.1 | 2017V-1085 | 4,038,943 |  | |  | |  |
| GCA_003311905.1 | Jul-25 | 3,832,137 |  | |  | |  |

**Table S3.** DNA sequences of the Classical and El Tor alleles of *ctxB* and *rstR*

| **Accession** | | **Strain name** | | **Serotype** | | **Biotype**  classical |
| --- | --- | --- | --- | --- | --- | --- |
| GCA_000621645.1 | | ATCC 14035(T) | | O1 | |  |
| ***ctxB1 s*equence** | | | | | | |
| ATGATTAAATTAAAATTTGGTGTTTTTTTTACAGTTTTACTATCTTCAGCATATGCACATGGAACACCTCAAAATATTACTGATTTGTGTGCAGAATACCACAACACACAAATACATACGCTAAATGATAAGATATTTTCGTATACAGAATCTCTAGCTGGAAAAAGAGAGATGGCTATCATTACTTTTAAGAATGGTGCAACTTTTCAAGTAGAAGTACCAGGTAGTCAACATATAGATTCACAAAAAAAAGCGATTGAAAGGATGAAGGATACCCTGAGGATTGCATATCTTACTGAAGCTAAAGTCGAAAAGTTATGTGTATGGAATAATAAAACGCCTCATGCGATTGCCGCAATTAGTATGGCAAATTAA | | | | | | |
| **rstR1 sequence** | | | | | | |
| ATGTTTAGTTCAAAAATTAGGGATTTAAGAGTTGAGAGAGATCTAAACCAAGAAGAAGTAGCAAATGGTATCGGCGTTGGAAAAAATACCTATTTAGCTTATGAAAAAGGCACACAATCACCAAAACTGGAAACTGTAGAAAAATTAGCAAAATTCTATGGTGTACCAATAGCTGAACTTGTCAGCGATAGCGAAACAAACATTGACGAAAAGCTGAAATCGAAAATCCGAATGATTGAATCACTTGATGAACCAGAAAAAGAGTCATTATTCATTTTGATGGAGGCTTTGCTGATGAGAAGTAAGAGTCGAGAAATACAAAAAGAATTTAGGTAG | | | | | | |
| **Accession** | **Strain name** | | **Serotype** | | **Biotype**  El Tor | |
| GCA_000006745.1 | N16961 | | O1 | |  |  |
| ***ctxB2* sequence** | | | | | | |
| ATGATTAAATTAAAATTTGGTGTTTTTTTTACAGTTTTACTATCTTCAGCATATGCACATGGAACACCTCAAAATATTACTGATTTGTGTGCAGAATACCACAACACACAAATATATACGCTAAATGATAAGATATTTTCGTATACAGAATCTCTAGCTGGAAAAAGAGAGATGGCTATCATTACTTTTAAGAATGGTGCAATTTTTCAAGTAGAAGTACCAGGTAGTCAACATATAGATTCACAAAAAAAAGCGATTGAAAGGATGAAGGATACCCTGAGGATTGCATATCTTACTGAAGCTAAAGTCGAAAAGTTATGTGTATGGAATAATAAAACGCCTCATGCGATTGCCGCAATTAGTATGGCAAATTAA | | | | | | |
| **rstR2 sequence** | | | | | | |
| ATGATTAAATTAAAATTTGGTGTTTTTTTTACAGTTTTACTATCTTCAGCATATGCACATGGAACACCTCAAAATATTACTGATTTGTGTGCAGAATACCACAACACACAAATATATACGCTAAATGATAAGATATTTTCGTATACAGAATCTCTAGCTGGAAAAAGAGAGATGGCTATCATTACTTTTAAGAATGGTGCAATTTTTCAAGTAGAAGTACCAGGTAGTCAACATATAGATTCACAAAAAAAAGCGATTGAAAGGATGAAGGATACCCTGAGGATTGCATATCTTACTGAAGCTAAAGTCGAAAAGTTATGTGTATGGAATAATAAAACGCCTCATGCGATTGCCGCAATTAGTATGGCAAATTAA | | | | | | |
|  | | | | | | |

**2. Figure(s)**


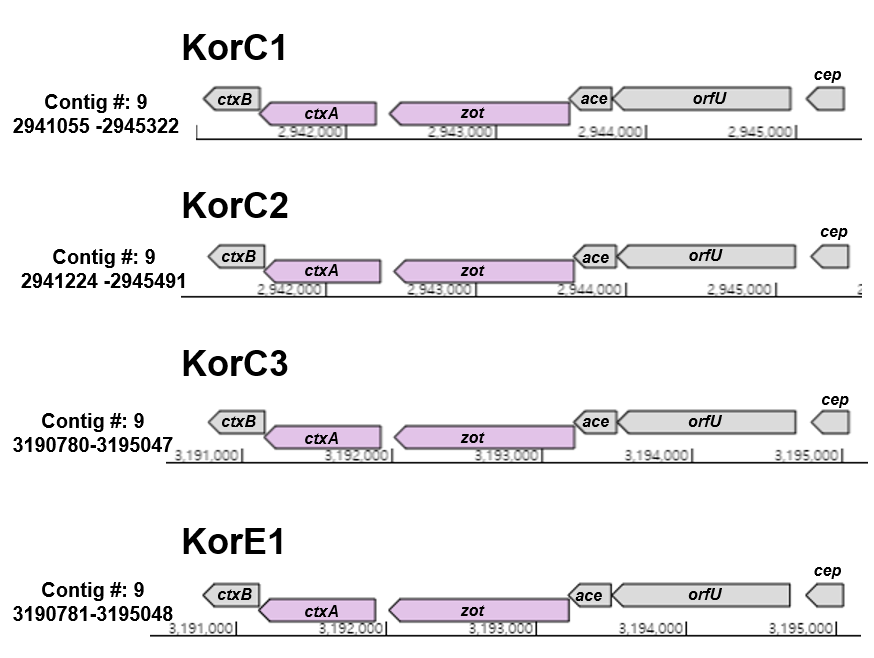


**Figure S1.** CTXφ prophage region found in all Korean isolates
